# A New Pterosaur (Pterodactyloidea: Azhdarchidae) from the Upper Cretaceous of Morocco

**DOI:** 10.1371/journal.pone.0010875

**Published:** 2010-05-26

**Authors:** Nizar Ibrahim, David M. Unwin, David M. Martill, Lahssen Baidder, Samir Zouhri

**Affiliations:** 1 School of Medicine and Medical Science, University College Dublin, Dublin, Republic of Ireland; 2 School of Museum Studies, University of Leicester, Leicester, United Kingdom; 3 School of Earth and Environmental Sciences, University of Portsmouth, Portsmouth, United Kingdom; 4 Laboratoire de Géosciences, Université Hassan II, Casablanca, Morocco; Raymond M. Alf Museum of Paleontology, United States of America

## Abstract

The Kem Kem beds in South Eastern Morocco contain a rich early Upper (or possibly late Lower) Cretaceous vertebrate assemblage. Fragmentary remains, predominantly teeth and jaw tips, represent several kinds of pterosaur although only one species, the ornithocheirid *Coloborhynchus moroccensis*, has been named. Here, we describe a new azhdarchid pterosaur, *Alanqa saharica* nov. gen. nov. sp., based on an almost complete well preserved mandibular symphysis from Aferdou N'Chaft. We assign additional fragmentary jaw remains, some of which have been tentatively identified as azhdarchid and pteranodontid, to this new taxon which is distinguished from other azhdarchids by a remarkably straight, elongate, lance-shaped mandibular symphysis that bears a pronounced dorsal eminence near the posterior end of its dorsal (occlusal) surface. Most remains, including the holotype, represent individuals of approximately three to four meters in wingspan, but a fragment of a large cervical vertebra, that probably also belongs to *A*. *saharica*, suggests that wingspans of six meters were achieved in this species. The Kem Kem beds have yielded the most diverse pterosaur assemblage yet reported from Africa and provide the first clear evidence for the presence of azhdarchids in Gondwana at the start of the Late Cretaceous. This, the relatively large size achieved by *Alanqa*, and the additional evidence of variable jaw morphology in azhdarchids provided by this taxon, indicates a longer and more complex history for this clade than previously suspected.

## Introduction

**Figure 1:**
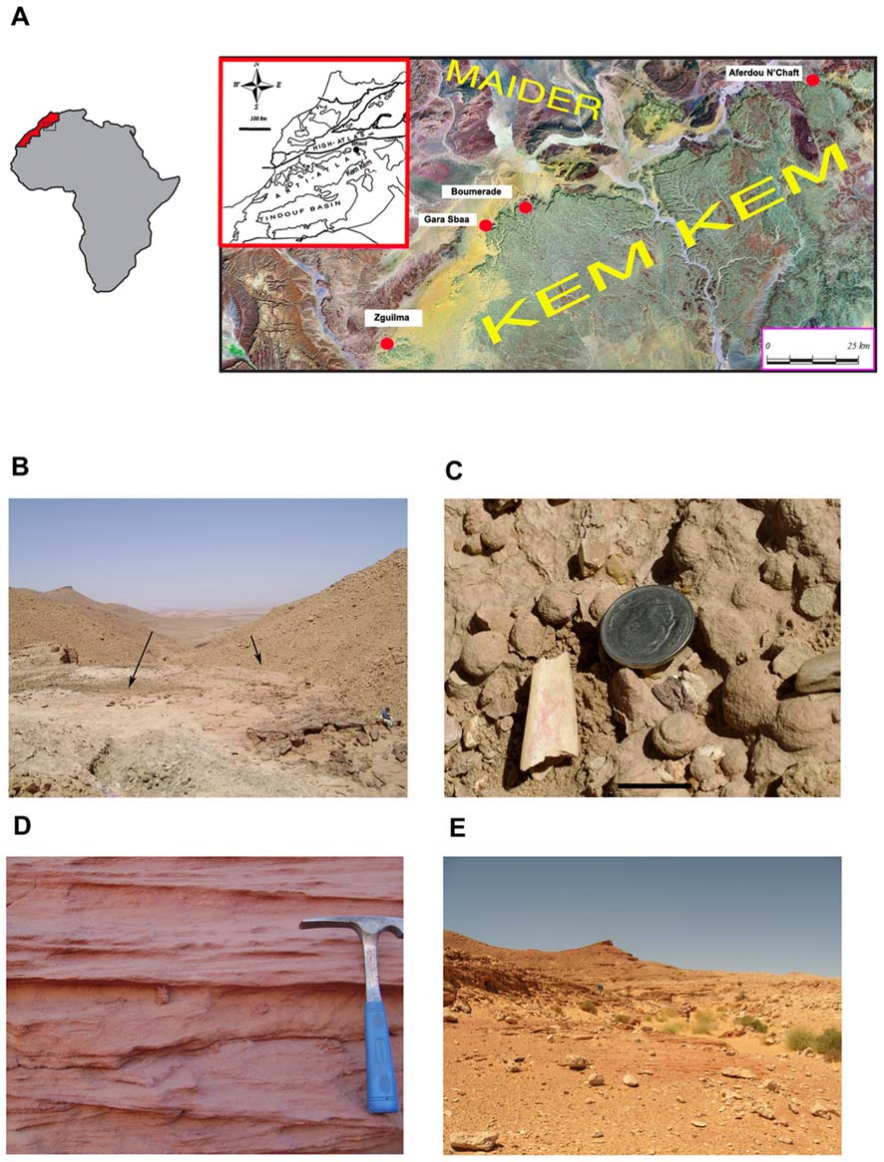
Pterosaur localities in the Kem Kem region of South Eastern Morocco. A) Map of Africa and satellite map of the Hamada Kem Kem and Maider region showing principal pterosaur localities. Inset: location of Kem Kem in Morocco. B) Boumerade locality (30° 32 ′49.00″ N 4° 42′ 55.45″ E), which includes most of the lower unit of the Kem Kem beds and just over one third of the upper unit (Sereno et al. 1996). Fossils were collected from areas marked by arrows, consisting of a sandstone matrix in a ∼2 m thick horizon. C) Pterosaur jaw tip found at Boumerade, scale bar 2 cm. D) Typical red sandstone cross bedding, found at the Aferdou N'Chaft (30° 53′ 51.23″ N 3° 52′ 13.42″) locality for example. E) Surface collecting in the sandstone outcrops of the Gara Sbaa locality (30° 30′ 40.64″ N 4° 50 42.87″ E).

The pterosaur fossil record from Africa is one of the least known for any continental land mass. This can largely be attributed to poor sampling – a consequence of the remoteness of many localities and political instability in several Saharan and sub-Saharan countries. With the exception of the pterosaur assemblage from the Upper Jurassic to Lower Cretaceous Tendaguru beds of Tanzania [1]–[3], there have been remarkably few finds in the southern half of the continent [4], [5], and several of these [6], [7] have yet to be verified (Yates pers comm. 2008). The northern half has yielded a greater array of pterosaur localities [8]–[13], with the single most important finds from the early Upper Cretaceous Kem Kem beds of Morocco (Figure 1, Table 1).

**Table 1:** Synopsis of pterosaur material described from the Kem Kem beds.

Specimen ID	Material	Taxonomic ID	Locality	Reference
Unknown	Single cervical vertebra	?Azhdarchidae	Unknown (‘Region of the Hamada du Guir, Taouz’)	[25]
LINHM 007	Single tooth	Ornithocheiridae	Unknown (‘West of the Hamada du Guir’)	[64]
BSP 1993 IX 338	Anterior section of rostrum	Azhdarchidae, *Alanqa saharica* nov. gen. nov. sp.	Unknown (‘East of Taouz’)	[15]
BSP 1996 I 36	Anterior section of mandibular symphysis	Azhdarchidae, *Alanqa saharica* nov. gen. nov. sp.	Unknown (‘East of Taouz’)	[15]
BSP 1997 I 67	Anterior portion of mandibular symphysis	Tapejaridae gen. et sp. Indet	Unknown (‘East of Taouz’)	[15]
BSP 1993 IX 4, 590-596	35 isolated teeth	Ornithocheiridae	Unknown (‘East of Taouz’)	[15]
LINHM 016 (holotype)	Anterior section of rostrum with dentition	Ornithocheiridae, *Coloborhynchus moroccensis*	Unknown, (‘near Begaa’)	[16]
MN 7054- V	Anterior section of ?rostrum	?Pteranodontidae	Unknown (‘Red Beds of the Hamada du Guir’)	[22]
FSAC- KK 26 (holotype)	Almost complete mandibular symphysis	Azhdarchidae, *Alanqa saharica* nov. gen. nov. sp.	Aferdou N'Chaft, near Begaa	This study
FSAC-KK 31	Section of mandibular symphysis	Azhdarchidae, *Alanqa saharica* nov. gen. nov. sp.	Boumerade	This study
FSAC-KK 27	Short section of rostrum	Azhdarchidae *Alanqa saharica* nov. gen. nov. sp.	Aferdou N'Chaft, near Begaa	This study
FSAC- KK 34	Incomplete cervical vertebra	Azhdarchidae	Aferdou N'Chaft, near Begaa	This study

Abbreviations: BSP, Bayerische Staatssammlung für Paläontologie und Geologie, Munich, Germany; FSAC, Faculté des Sciences Ain Chock, Université Hassan II, Casablanca, Morocco; LINHM, Long Island Natural History Museum, MN, Museu Nacional/UFRJ, Rio de Janeiro, Brazil; TMM, Texas Memorial Museum, Austin, Texas, USA.

Several clades of pterosaur have been reported from the Kem Kem beds. Ornithocheirids are represented by numerous finds of long, slender, variably recurved teeth [14], [15] and a fragmentary rostrum, the holotype of *Coloborhynchus moroccensis* [16]. The similarity of this fossil to more complete remains of coloborhynchids including *C*. *clavirostris* [17] and *C*. *robustus* [18], leaves no doubt as to its identification as an ornithocheirid [e.g., 19].

**Figure 2:**
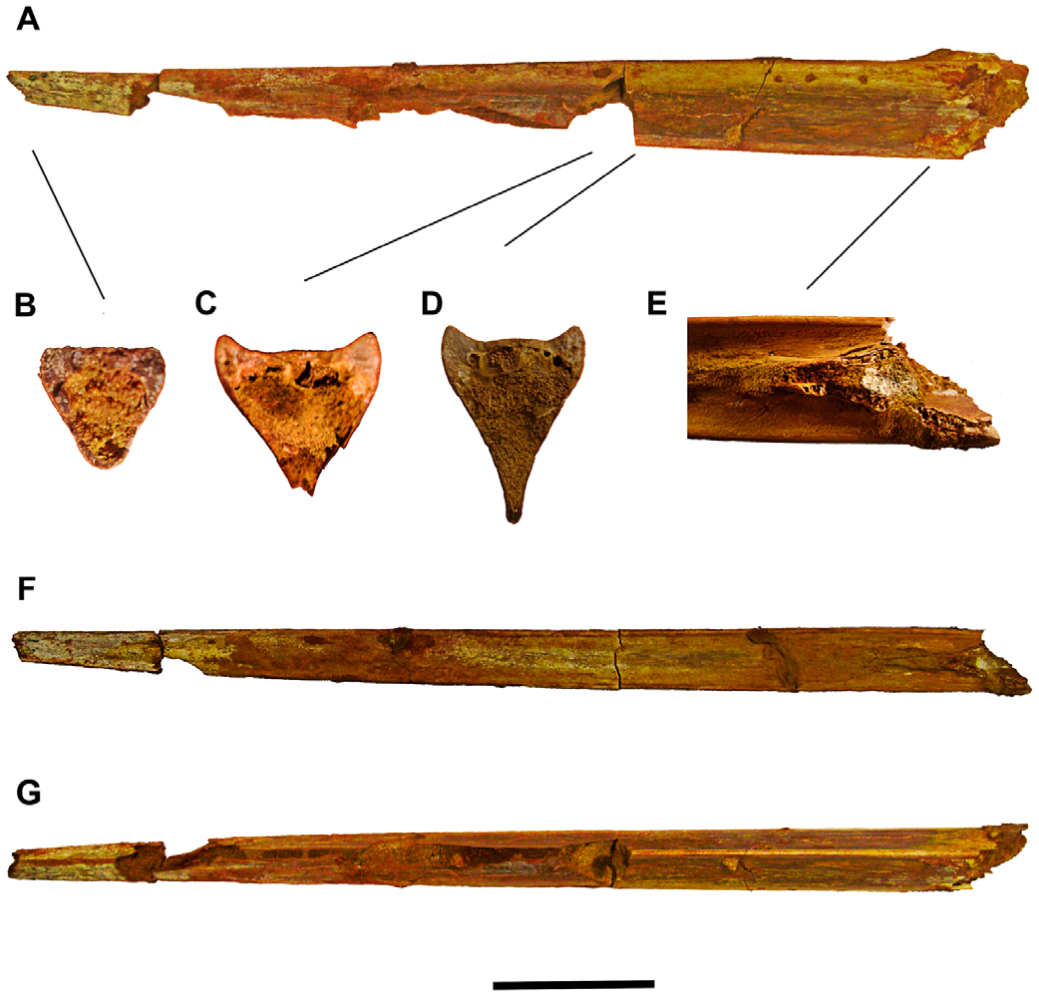
Holotype specimen (FSAC-KK 26) of *Alanqa saharica* nov gen. nov sp. A) lateral view, F) dorsal view and G) ventral view. Cross sections B, C and D (not to scale) showing internal morphology of the three fragments. E) Details of the posterior end of the mandibular symphysis in dorsal view. Scale bar for A, F, G: 5 cm.

A fragment of an edentulous jaw was tentatively identified as an anterior portion of the rostrum of a pteranodontid [15]. However, it does not exhibit distinctive features of this pterosaur, such as a pronounced curvature of the jaws when seen in lateral view ([20], Figure 2), and it has been proposed instead [21] that this find be assigned to Azhdarchidae, an idea that we support here. A second fragment of an edentulous jaw, interpreted as part of a mandibular symphysis [22] has also been tentatively identified as pteranodontid. It too lacks any characters unique to this clade, and its identity remains uncertain.

**Figure 3:**
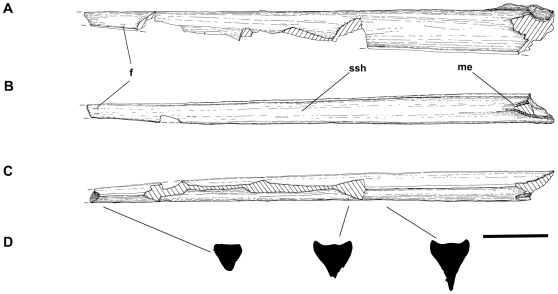
Line drawing of the mandibular symphysis of *Alanqa saharica* nov gen. nov sp. (FSAC-KK 26). A) lateral view, B) dorsal view, C) ventral view, D) Cross sectional morphology. Abbreviations: f: foramina, ssh: symphyseal shelf, me: midline eminence. Scale bar: 5 cm.

Two distinct clades of azhdarchoids have been reported from the Kem Kem beds. Tapejarids are represented by a single jaw fagment, BSP 1997 I 67, bearing the remains of a prominent midline crest [15]. This fragment has generally been interpreted as part of the mandibular symphysis [15], [22], but might pertain to the rostrum, in which case it is worth noting that the jaw and crest morphology of the Kem Kem specimen is consistent with that reported for tapejarids ([23] fig. 3) and to some extent tupuxuarids ([24] fig. 3).

In the first report of a pterosaur from these deposits, Kellner and Mader [25] identified an elongate cervical vertebra as azhdarchid, but this specimen has yet to be illustrated and the assignment remains unverified. More recently, a slender, elongate edentulous fragment of a rostrum was tentatively assigned to Azhdarchidae by Wellnhofer and Buffetaut [15]. Averianov et al. [21] were less circumspect, positively identifying the specimen as azhdarchid, and went even further in identifying the ‘pteranodontid’ jaw fragment described by Wellnhofer and Buffetaut [15] as part of the mandibular symphysis, and the tapejarid jaw as a ‘posterior fragment of the premaxilla, with an anterior fragment of the cranial crest’. Other authors [26] seem to have adopted this interpretation.

In summary, the taxonomic identity and phylogenetic relationships of edentulous pterosaurs from the Kem Kem beds remain uncertain. The exact number of species: one, two, three, or more, is unclear; the assignment of specimens to these species is unclear; and the relationships of these species to more inclusive clades such as Pteranodontidae, Tapejaridae and Azhdarchidae is also unclear. A further complication concerns the lack of precise locality data for all specimens reported so far, as many of these have come from commercial sources.

In April and November to December 2008 we carried out expeditions to South Eastern Morocco and collected pterosaur remains from several different localities (Aferdou N'Chaft, Boumerade, Gara Sbaa, Zguilma; Figure 1), sometimes with the help of local villagers. Most of the discoveries were made at Boumerade and Aferdou N'Chaft, the latter yielding a remarkably well preserved and almost complete mandibular symphysis of a large edentulous pterosaur. This find, coupled with recent improvements in our understanding of pterosaur anatomy and phylogenetic relationships [19], [27], [28] permitted the identification and diagnosis of a new species of azhdarchid. This, in turn, provided important insights into the identity of other fragments of edentulous jaws previously reported from the Kem Kem beds and a better understanding of pterosaur evolution in Africa during the Cretaceous.

### Geology

The escarpment of the Hamada Kem Kem stretches for around 250 km along the Moroccan-Algerian border (Figure 1). It is part of the so called ‘Continental Intercalaire’ a term used by Lavocat [29], [30] to describe a wide array of deposits across North Africa [31] and spanning a considerable period of time. The term ‘Kem Kem beds’ was introduced by Sereno et al. [32] for the red beds of the ‘Continental Intercalaire’ cropping out in South Eastern Morocco along the border with Algeria (Figure 1). Direct lateral equivalents to the Kem Kem occur in Algeria (e.g., Gara Samani [33]). In South Eastern Morocco, outcrops of the Kem Kem sequence are found in different regions (see for example [34], [35]).

The Kem Kem beds record a complex fluvial system, the detailed structure of which is not yet fully understood. Most studies have focused on the vertebrate fauna [36], [37] and contain limited stratigraphical and sedimentological data [15], [34]. The Kem Kem beds lie unconformably upon folded Palaeozoic rocks [32] and are overlain by Cenomano-Turonian limestones that record a major marine transgression [38].

While there is general agreement that the Kem Kem beds lie within the late Lower to early Upper Cretaceous the exact age of this sequence remains uncertain. The vertebrate assemblages of the Kem Kem beds and those of the Bahariya Formation in Egypt, which is reliably dated as earliest Upper Cretaceous [39], are remarkably similar [10], [32], which has led most workers to favour a Cenomanian age for the Kem Kem [15], [32], [36], [40]. This is supported by the observation that several of the elasmobranch taxa found in the Kem Kem beds are also found in the Egyptian assemblage, including one species (*Serratolamna amonensis*) that is restricted to the Cenomanian [32].

Archosaur fossils occur in two main sedimentary units and consist mainly of disarticulated elements [32]. Isolated teeth of pterosaurs are fairly common, but other elements such as fragments of rostra, mandibular symphyses, cervical vertebrae and other postcranial bones are extremely rare. Three edentulous pterosaur jaw fragments were discovered at the locality of Boumerade (Figure 1), near Lake Maider in April 2008 and a further jaw fragment was found there in November 2008. A second locality, Aferdou N'Chaft, located near the village of Begaa and 10 km north-east of Taouz yielded a relatively complete mandibular symphysis (described below), two other edentulous jaw fragments, isolated teeth and part of a cervical vertebra (also described below). These remains were collected from a reddish brown layer of fine, loosely adhesive sandstone near the top of the sequence exposed at Aferdou N'Chaft. Taouz was mentioned as a possible locality by Wellnhofer and Buffetaut [15], but these authors did not provide precise locality information. It is possible that the material described in their paper also originates from Aferdou N'Chaft. The holotype of *Coloborhynchus moroccensis* [16], [41] was also reported as from a locality near the village of Begaa. However, the map illustrated by Mader and Kellner [16] incorrectly positions Begaa southwest of Taouz and close to Gara Sbaa, casting doubt on the accuracy of this locality information.

## Methods

All new specimens discussed in this paper were collected in South Eastern Morocco, from the localities described above and in the systematic section below. Comparative material housed in Germany, Morocco and the United States (see below) was examined first hand by three of us (NI, DU, DM). Specimens were hand prepared by one of us (NI) at University College Dublin.

## Results

### Systematic Paleontology

Pterosauria Kaup 1834 [42]

Pterodactyloidea Plieninger 1901 [43]

Azhdarchoidea Nesov 1984 [44] (sensu Unwin 2003 [19])

Azhdarchidae Nesov 1984 [44]

### *Alanqa* gen. nov

urn:lsid:zoobank.org:act:B6244462-2CDC-409A-91F7-5A5A1F1A99BC

#### Etymology

*Al Anqa* (Arabic) a Phoenix - like mythological flying creature from ancient times, similar to the Persian Simurgh.

#### Type species

*Alanqa saharica*, gen. et sp. nov.

#### Diagnosis

As for type species *A*. *saharica*.

### *Alanqa saharica*, gen. et sp. nov

urn:lsid:zoobank.org:act:19995B00-FFB3-4747-8A37-17EBFCA8B2B2

#### Etymology

*Sahara*, Desert (Arabic); -*ica,* belonging to (Greek). Named for the region and environment where the holotype was found.

#### Holotype

FSAC-KK 26 (Faculté des Sciences Ain Chock, Université Hassan II, Casablanca); almost complete mandibular symphysis.

#### Type locality

Aferdou N'Chaft, near Begaa, Province d'Errachidia, Morocco, 30° 53′

51.23″ N 3° 52′ 13.42″ E

#### Horizon

Kem Kem beds; Cenomanian, (Sereno et al. 1996).

#### Referred specimens

BSP 1996 I 36, anterior portion of mandibular symphysis from the Kem Kem beds ‘possibly near Taouz’ [15]; FSAC-KK 31, short section of mandibular symphysis, collected at Boumerade near the Maider lake, Province d'Errachidia, Morocco, 30° 32′ 49.00″ N 4° 42′ 55.45″ E; BSP 1993 IX 338, incomplete rostrum from the Kem Kem beds ‘possibly near Taouz’ [15]; FSAC-KK 27, anterior portion of rostrum, collected at Aferdou N'Chaft, near Begaa, Province d'Errachidia, Morocco, 30° 53′ 51.23″ N 3° 52′ 13.42″ E

#### Diagnosis

Elongate mandibular symphysis (length/maximum depth >10) with remarkably straight dorsal and ventral profile in lateral view and a well developed “V”-shaped midline ridge that bounds the posterior end of the occlusal surface of the mandibular symphysis, bifurcates posteriorly and projects well above the occlusal margin of the symphysis.

#### Material

**Figure 4:**
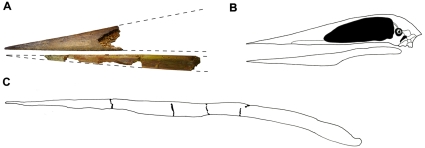
Reconstructed jaws of *Alanqa saharica* compared to other azhdarchids. A) Holotype specimen (mandibular symphysis) of *Alanqa saharica* nov gen. nov sp. (FSAC-KK 26) matched with one of the referred rostra, BSP 1993 IX 338. B) Skull outline of the azhdarchid *Zhejiangopterus linhaiensis*, modified from Unwin and Lü [58] and Witton and Naish [65]. C) Lower jaw of *Quetzalcoalus sp*., redrawn from Kellner and Langston, specimen TMM 42161-2 [45].

All five jaw fragments assigned here to *Alanqa saharica* are well preserved, uncrushed and, unlike the vast majority of pterosaur fossils, retain their original three dimensional shape. The holotype mandibular symphysis (FSAC-KK 26, Figures 2 and 3, Table 2), identified as such by its similarity to the mandibular symphysis of *Quetzalcoatlus* sp. ([45] Figure 4A, 4C), was preserved in a soft crumbly sandstone that, apart from small isolated patches that strongly adhere to the bone surface, was easily removed. The holotype consists of three contiguous orange-brown fragments with a combined length of 344 mm. The anterior tip of the symphysis is missing: extending the dorsal and ventral jaw margins, or the lateral margins of the jaw, forward to the point where they meet, which is consistent with jaw shape in other azhdarchids such as *Zhejiangopterus linhaiensis* [46] and *Quetzalcoatlus* sp. [45] indicates that the length of the missing portion may have been up to 80 mm. Much of the ventral half of the intermediate fragment is broken away, especially anteriorly where a small part of the palatal surface is also missing. The symphysis appears to have fractured at its posterior end where the mandible diverged into separate rami.

**Table 2:** Principal measurements of pterosaur jaw fragments and part of a cervical vertebra from the Kem Kem beds of Morocco.

Jaw remains	Preserved length	Widest point of palate/symphyseal shelf	Maximum height (lateral view)	Angle in degrees
FSAC-KK 26	344	20	33	7°
FSAC-KK 27	132	12	22	10°
FSAC-KK 31	73	17	Damaged	Damaged
**Cervical vertebra**	**Length**	**Width**		
FSAC-KK 34	55	83		

Measurements in mm.

A second fragment (FSAC-KK 31) from Boumerade is 73 mm in length and represents an anterior portion of the mandibular symphysis. The specimen is creamy coloured, the tip of the mandibular symphysis is missing and much of the dorsal surface is broken away. Comparison with the holotype suggests that this individual was slightly larger than that represented by FSAC-KK 26.

A third fragment, BSP 1996 I 36, described by Wellnhofer and Buffetaut [15] compares almost exactly to the anterior portion of FSAC-KK 26 (matched on the basis of the cross-sectional profile of the mandible which, in *A. saharica*, varies along the symphysis, as described below) and would appear to represent an individual of about the same size as the holotype.

An incomplete but well preserved rostrum (BSP 1993 IX 338), first described by Wellnhofer and Buffetaut [15] and originally identified as possibly pteranodontid, is reidentified here as the rostrum of *A. saharica*. In terms of its shape and breadth the palatal surface of this fragment matches closely to the anterior portion of the holotype, and it would seem that these specimens represent individuals of similar size. A second rostral fragment (FSAC - KK 27) is similar in shape and size to BSP 1993 IX 338, but is less complete and also lacks the tip of the rostrum.

#### Description

The mandibular symphysis of *A. saharica* which, presumably, consists solely of the fused dentaries, is highly elongate with a length to depth index of between 10 and 11, depending on the true length of the missing anterior portion. This value is among the highest for edentulous pterosaurs and only surpassed by *Quetzalcoatlus* sp. (>14) which has an exceptionally elongate mandibular symphysis [45]. Seen in lateral view both the dorsal and ventral margins of the symphysis are remarkably straight. They converge evenly and at a very low angle (7 degrees) which, extrapolated forward, results in a sharp tipped, lance-shaped symphysis. This restoration is consistent with the evenly tapered profile of the mandibular symphysis in *Zhejiangopterus linhaiensis* [46] and *Quetzalcoatlus* sp. [45].

Toward the anterior tip of the symphysis the lateral surfaces are more or less flat and the cross-section of the jaw is triangular with rounded corners (Figure 2B). Posteriorly the upper half of the symphysis widens more rapidly than the lower half which forms a long low blade-like ventral keel extending almost the entire length of the symphysial part of the mandible. At about its mid–point (Figure 2C), when seen in cross-section, the dorsal half of the symphysis is swollen outwards while the lower half forming the keel is pinched inward. This cross-sectional shape, which takes the form of a fat ‘Y’, is further emphasised at the posterior end of the symphysis (Figure 2D) where the keel is even more pinched, so that the symphysis forms a relatively deep, narrow structure. This is underscored by comparison of width∶ height ratios along the jaw which change from 1∶1 at the anterior end to 1∶1.45 at the posterior end.

Posteriorly, the symphyseal shelf is bounded on either side by robust dental margins consisting of a dorsally directed ridge of bone about two millimetres in depth and terminating in a narrow, but slightly rounded apex (Figures 2A, E). In cross-section (Figures 2C, D) the profile of the dental margin is asymmetric with an almost vertical, but rounded, lateral surface that contrasts with the flat, or slightly concave, inwardly sloping medial surface. The dental margins slowly decline in size anteriorly and eventually fade out altogether such that the anterior 53 mm of the preserved portion of the symphysis is essentially flat (Figure 2B).

While the symphyseal shelf is flat anteriorly, proceeding posteriorly its middle portion becomes gently convex, forming a low midline swelling (Figure 2C). Toward the posterior end of the mandibular symphysis this convexity becomes slightly more pronounced and extends across the entire shelf (Figure 2D). A convex surface is typical of rostra, rather than the mandibular symphysis, but does occur in the latter as seen, for example, in ornithocheirids from the Cambridge Greensand of England [47].

At the posterior end of the symphysis the shelf develops into a distinctive midline eminence that projects above the profile of the dental margins and is about three millimetres in height (Figure 2E). This ‘V’-shaped structure, the apex of which points anteriorly, bifurcates at a low angle (10° initially, widening to about 27°) into posterolaterally trending narrow ridges of bone that, judging from their orientation, must eventually have merged with the dorsal margin of each mandibular ramus. A similar eminence is present in *Bakonydraco galaczi* [48], but located at about the mid-length of the symphysis rather than at its posterior end. A narrow medial opening preserved between these ridges appears to correspond to a similar opening reported in *Quetzalcoatlus* sp. [45] which, in addition to transmitting blood vessels and nerves, may have permitted pneumatisation of the symphysis.

The external surfaces of the symphysis bear several small, slit-like foramina aligned roughly parallel to the long axis of the jaw. These foramina, irregularly distributed on the lateral surfaces of the symphysis, but arranged in pairs on the palatal surface, are connected to a system of fine grooves dispersed over the surface of the jaw. Presumably, these housed blood vessels and nerves that supplied the rhamphothecae covering the jaws [49]. First noted by Wellnhofer and Buffetaut [15] in *Alanqa*, these features, sometimes found in other azhdarchoids although in a less developed state, are typical of azhdarchids [e.g., 21,44,50], especially the paired foraminae on the palate [15], [21], [44] which may be diagnostic for this clade.

Details of the internal structure of the symphysis are exposed in all three specimens. These show (Figure 2C) that the walls of the symphysis were composed of remarkably thin sheet of bones approximately 0.3 to 1.0 mm in thickness. Apart from occasional struts and bars of bone, the remainder of the symphysis appears to have been hollow, although a ‘foam-like’ infilling of cancellous bone was present in the ridge bounding the posterior end of the palatal surface (Figure 2E).

Establishing the original dimensions of *A*. *saharica* on the basis of such fragmentary remains is difficult. In *Quetzalcoatlus* sp., seemingly the taxon most similar to *A*. *saharica* (see below), the symphysis forms approximately 60% of the total length of the mandible [45]. Assuming that similar proportions applied to *A*. *saharica* this indicates an original mandible length for the holotype of approximately 0.675 m. Since the symphysial portion of the mandible is proportionately longer in *Quetzalcoatlus* sp. than in other azhdarchids (in *Zhejiangopterus linhaiensis* it forms 58% of the mandible, in *Bakonydraco galaczi* only 45%), the predicted mandible length can be taken as a minimum likely size for the holotype.

#### Assignment of referred specimens

In its general morphology FSAC-KK 31 compares closely to the holotype. Most importantly, the profile in lateral view and the cross-sectional profile, both of which are distinctive for *Alanqa saharica*, are the same in the holotype and in FSAC-KK 31. The same arguments apply to BSP 1996 I 36. The lateral and cross-sectional profiles of this specimen ([15]) are identical to those of FSAC-KK 26 and FSAC-KK 31.

Assignment of the two fragmentary rostra (BSP 1993 IX 338, FSAC-KK 27) to *A*. *saharica*, first mooted by Averianov et al. [21] for the former specimen, is based on the observation that the shape and size of the palatal surface shows an almost perfect match with that of the mandibular symphysis FSAC-KK 26. In addition, the rostral fragments exhibit a feature, the presence of paired foramina on the palatal surface, that is typical for azhdarchids, and since only a single azhdarchid is known from the Kem Kem beds it is highly likely that they belong to this taxon. It is also noteworthy that a restoration of the jaws of *A*. *saharica* (Figure 4), shows a remarkably good match, in terms of the relative depths and shapes of the rostrum and mandibular symphysis, to the jaws of *Zhejiangopterus linhaiensis* [46] and *Quetzalcoatlus* sp. (Habib, pers. comm. 2010).

#### Comparison

The complete absence of tooth alveoli on the dental margins of the five specimens assigned to *Alanqa saharica*, which represent all but the anteriormost tip of the mandibular symphysis and much of the rostrum, show that this taxon was edentulous. Edentulous forms are found in two clades of pterosaurs: Pteranodontia (*Pteranodon*, Nyctosauridae) and Azhdarchoidea (Tapejaridae, Chaoyangopteridae, Thalassodromidae and Azhdarchidae).

*A*. *saharica* can be safely excluded from Pteranodontia in that all pteranodontians have mandibular symphyses whose profile, seen in lateral view, shows a much more rapid increase in depth posteriorly than in *A*. *saharica*, and is as deep, or deeper than the rostrum at any corresponding point along the jaws. By contrast, the rostrum of *A*. *saharica* is always deeper than the corresponding portion of the mandibular symphysis. In the same profile, the degree of curvature of both the mandibular symphysis and the rostrum of pteranodontians [20], [51], [52] is markedly greater than in *A*. *saharica*. The degree of curvature evident in pteranodontians varies, possibly due in part to variable compaction during burial, but in all examples examined during this study some degree of curvature was evident and not one exhibited the remarkable straightness of the mandibular symphysis evident in *A*. *saharica*. The latter is further distinguished from all pteranodontians by the Y-shaped cross-sectional profile of the mandibular symphysis, which is quite unlike the triangular profile found, for example, in *Pteranodon longiceps* [20]. In addition, the profile of the mandibular symphysis, in palatal aspect, of *P. longiceps*, is distinctly tapered [20], while that of *A*. *saharica* is of more or less even width for much of its length (Figure 3B). The jaws of pteranodontians also appear to lack the prominent paired foramina present on the palatal surfaces of the rostrum and mandibular symphysis of *A*. *saharica* and other azhdarchids.

*Alanqa saharica* can also be safely excluded from Tapejaridae (sensu Lü et al. [23]). These pterosaurs are characterised by short, deep jaws the anterior ends of which are ventrally deflected [23], [53]. These features are not present in *A*. *saharica* and this pterosaur also lacks the short deep mandibular crest and tall premaxillary crest that are typical of tapejarids [23], [53], [54].

The elongate jaws of *A*. *saharica* show much greater similarity to those of long-snouted azhdarchoids, including Chaoyangopteridae, Thalassodromidae and Azhdarchidae, than to other edentulous pterosaurs. While the lateral profile of the rostrum of *A*. *saharica* compares closely to that of chaoyangopterids [27], the long, straight, lance-shaped mandibular symphysis is distinctly different from the shorter, deeper, gently curved lower jaw of, for example, *Chaoyangopterus zhangi* [55] and *Shenzhoupterus chaoyangensis* [27]. Moreover, in at least some chaoyangopterids, such as *Lacusovagus magnificens*, the jaws are proportionately much wider than in *A*. *saharica* and have a different profile in palatal aspect: the anterior half of the rostrum is tapered while the posterior half is of even width [56].

*Alanqa saharica* shares four unique features in common with neoazhdarchians (Thalassodromidae + Azhdarchidae): seen in lateral view the rostrum is always deeper than the mandibular symphysis at any corresponding point along the jaws ([24], [46], [53], Habib pers. comm. 2010); the mandibular symphysis is long, straight and low (Figures 2, 3; [24], [45], [53]); the mandibular symphysis has a low, elongate, blade-like ventral keel; and the cross-sectional profile of the mandibular symphysis is triangular near the tip of the jaw, but becomes Y-shaped posteriorly.

Several unusual morphological features support the assignment of *A*. *saharica* to Azhdarchidae, rather than Thalassodromidae. The mandibular symphysis is extremely elongate, with a length/depth ratio that exceeds ten, a value that occurs in some azhdarchids, such as *Quetzalcoatlus* sp. [45], but not in thalassodromids, where the symphysis is proportionately shorter and deeper [24]. As in other azhdarchids, including *Azhdarcho lancicollis* [44], *Bakonydraco galaczi* [48] and *Volgadraco bogolubovi* [21], the palatal surface of both the rostrum and mandibular symphysis bear paired foramina. This arrangement of foramina seems to be absent in thalassodromids and other azhdarchoids. Finally, *A*. *saharica* lacks the development of the anterior portion of the mandibular symphysis into a narrow blade-like structure, with a sharply ridged palatal surface, as reported in *Thalassodromeus sethi* [57].

Among azhdarchids, the jaws of *A*. *saharica* most closely resemble those of *Quetzalcoatlus* sp., especially when compared in lateral view (Figure 4A,4C). Nonetheless, two features distinguish these taxa. The mandibular symphysis of *Quetzalcoatlus* sp. is relatively much more elongate than that of *A*. *saharica*. *Quetzalcoatlus* sp. lacks the raised, V-shaped midline structure that marks the posterior end of the palatal surface of the mandibular symphysis in *A*. *saharica*.

Other azhdarchids in which remains of the jaws are preserved include *Azhdarcho lancicollis* [44], *Zhejiangopterus linhaiensis* [46], [58], *Bakonydraco galaczi* [48] and *Volgadraco bogolubovi* [21].

As previously noted, in their general morphology jaw fragments assigned to *A. lancicollis* ([44] pl.VII, figs. 10, 11) closely resemble those assigned here to *A*. *saharica*. Detailed comparison is hindered by the incompleteness of the jaw fragments of *A*. *lancicollis*, however, those that can be assigned to the rostrum bear a narrow, midline ridge on the palatal surface, (DMU pers obs), a feature that seems to be absent in *A*. *saharica*.

*Zhejiangopterus linhaiensis*, the only azhdarchid known from relatively complete skull material [46], [58], also has jaws that are generally similar to those of *A*. *saharica* (Figure 4). However, the mandibular symphysis of *A*. *saharica* is much more elongate (L/D >10) than that of *Z. linhaiensis* (L/D <8). The symphysis of the latter has a more rounded ventral profile and appears to lack the eminence that, in *A*. *saharica*, bounds the posterior end of the symphysis and projects above the occlusal margin of the lower jaw.

The mandibular symphysis of *Bakonydraco galaczi* ([48], fig 2a, b) is strikingly different from that of *A*. *saharica* with a much shorter and deeper profile in lateral view and a ventral keel with an apex located at approximately the mid-length point of the symphysis. Posterior to this point the ventral keel is grooved. In addition, the mandibular symphysis is relatively much broader than in *A*. *saharica* and, in dorsal view, tapers from the anterior tip to the mid-length point of the symphysis rather than along its entire length.

*Volgadraco bogolubovi* is known from a single jaw fragment interpreted as part of the mandibular symphysis [21]. Assuming this is correct, then *V. bogolubovi* is distinguished from *A*. *saharica* by the gentle curvature of the symphysis in lateral view, which is absent in *A*. *saharica*, and the V-shaped cross section of the symphysis which is quite unlike the Y-shape seen in *A*. *saharica*.

### Azhdarchidae gen. et sp. indet

#### Material

**Figure 5:**
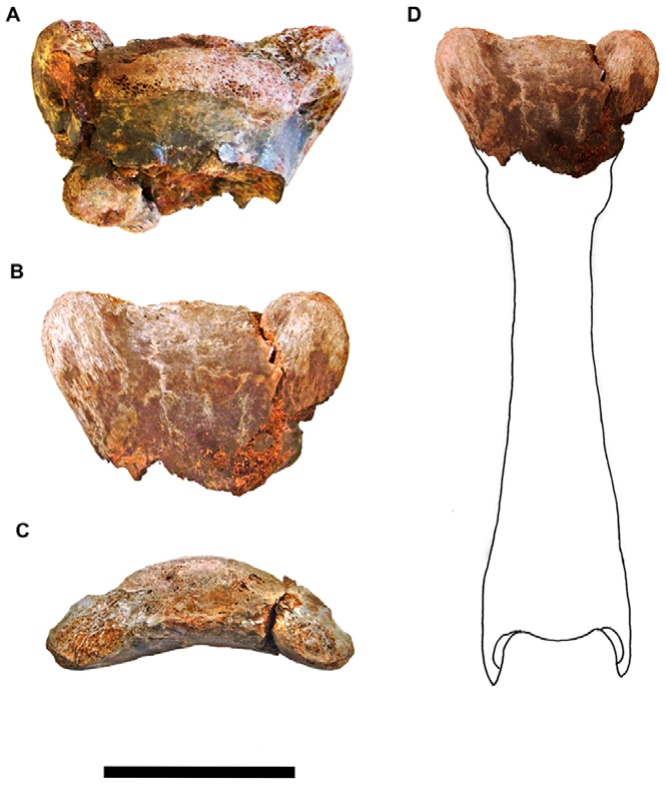
Posterior end of azhdarchid cervical vertebra (FSAC-KK 34). A) dorsal, B) ventral and C) posterior view. D) missing parts reconstructed and redrawn using images and drawings in Ösi et al ([48] figures 4 and 5) and Buffetaut et al. ([59] figure 2). Scale bar: 5 cm.

**Figure 6:**
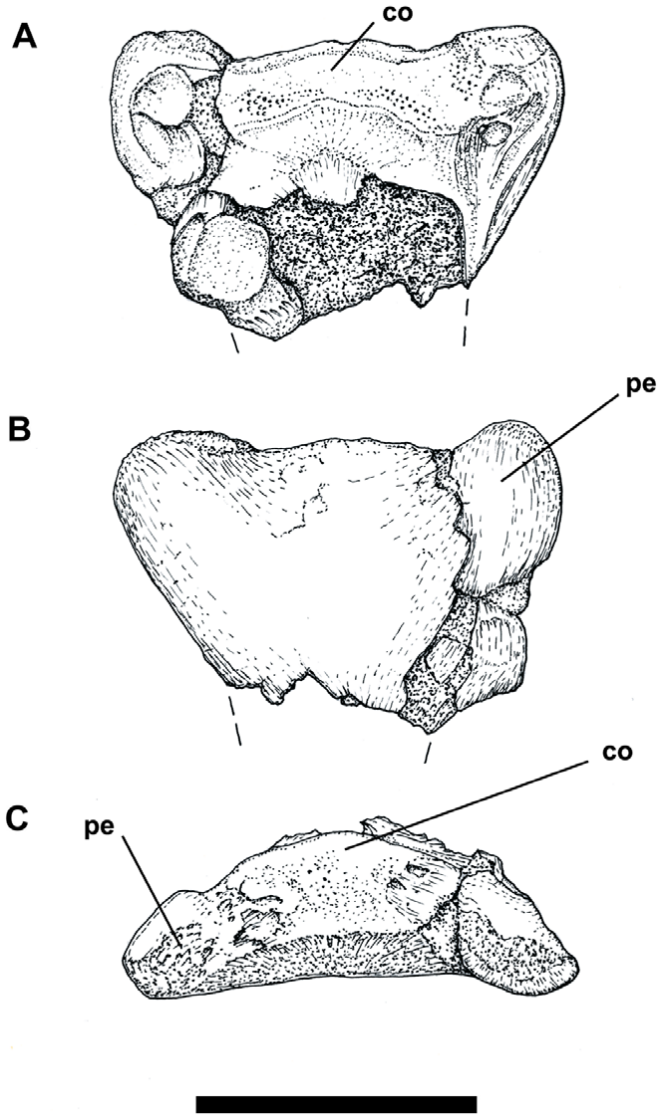
Drawing of FSAC-KK 34. A) dorsal, B) ventral and C posterior view. Abbreviations: co, condyle; pe, postexapophysis Scale bar: 5 cm.

A fragment of the posterior termination of a mid-series cervical vertebra (FSAC-KK 34); (Figures 5 and 6, Table 2).

#### Locality

Aferdou N'Chaft, near Begaa, Province d'Errachidia, Morocco, 30° 53′ 51 23″ N 3° 52′ 13 42″ E.

#### Horizon

Kem Kem beds, Cenomanian [32].

#### Preservation

The reddish-grey fragment, a cervical vertebra condyle bearing large postexapophyses, retains its original three dimensional shape and bears fine surface detail. The left postexapophysis has been fractured and subsequently repaired. In comparison with other more complete remains of cervical vertebrae of *Quetzalcoatlus* sp., *Azhdarcho lancicollis* and *Zhejiangopterus linhaiensis* and other azhdarchids [44], [45], [58], [59], the dorsoventral flattening of the condyle and the well developed postexapophyses indicate that this fragment likely pertains to a mid-series cervical. A more precise identification is not possible.

#### Description

The articular surface of the condyle caps the posterior termination of a short section of the centrum, which projected well posterior to the neural arch and postzygapophyses, as is typical for pterosaur cervical vertebrae. The condyle itself was broad and low, with a marked dorsoventral flattening. Seen in dorsal view, the condyle has a gently convex posterior profile, the articular surface extending onto the lateral surfaces of the centrum on either side. The anterior margin of the articular surface is gently embayed medially, as is typical for azhdarchid cervicals. Lateral regions of the dorsal aspect of the articular surface are intensively pitted, more so on the right side. In posterior view the condyle has an even, convex profile which slopes ventrally on either side into the bases of the postexapophyses. The ventral margin of the articular surface lies at about mid-height and is rather uneven.

The ventral surface of the centrum is remarkably flat, curving dorsally between the postexapophyses posteriorly to meet the ventral margin of the articular surface. The postexapophyses consist of well developed tongue-shaped posterolaterally directed bony projections originating from the posterolateral corners of the centrum. In posterior view each postexapophysis is directed laterally and slightly ventrally and is separated from the condyle by a shallow sulcus. The ventral surface of each postexapophysis bears distinct muscle scarring with a lineation directed toward the posterior margin. The outline of each postexapophysis, in dorsal, or ventral aspect, appears slightly different: that of the right side is more rounded, that on the left more elongate, although this asymmetry might be due in part to preservation.

The preserved width of this specimen, measured across the postexapophyses, is 83 mm. In examples of *Quetzalcoatlus* sp. the comparable measurement reaches a maximum of approximately 70 mm [60]. This suggests that the individual represented by this Kem Kem specimen likely achieved a wingspan in excess of the five meters estimated for *Q*. sp.

#### Comparison

In general, mid-series cervicals of *Quetzalcoatlus* sp. *Azhdarcho*, *Phosphatodraco* and *Arambourgiania*, together with more fragmentary remains from Russia [50], [61] and Senegal [8], appear to have articular condyles that are relatively higher and more convex when seen, for example, in posterior view. However, some cervicals of *Quetzalcoatlus* sp. also exhibit the flattened condition [60], [62] evident in the Kem Kem specimen. Thus this variation may be related to position within the cervical series, rather than being of any taxonomic significance. Notably, a relatively short azhdarchid cervical attributed to *Bakonydraco* also has a flattened condyle and well developed postexapophyses, as in *Alanqa*, but is distinguished from the latter by the presence of a pneumatic opening piercing the centrum beneath the articular condyle ([48] fig 5 A_8_).

#### Other azhdarchid material from the Kem Kem Beds

In addition to the jaw remains assigned to *Alanqa* and the fragmentary cervical, described above, several additional fossils from the Kem Kem beds have been identified as azhdarchid. These include a complete mid-series cervical [25], a large humerus, another cervical vertebra and a fragmentary rostrum [63]. We are also aware of further undescribed specimens including a well preserved cervical vertebra that appear to be azhdarchid. *Alanqa* is still insufficiently well known for us to be able to demonstrate that some, possibly all, of this postcranial material belongs to this pterosaur, thus no formal assignments are made here. However, in the absence of evidence to the contrary, we think it reasonable to assume that all these azhdarchid remains likely pertain to *Alanqa*.

## Discussion

### The Kem Kem pterosaur assemblage

The new finds reported here confirm the existence of an azhdarchid pterosaur in the Kem Kem assemblage and are sufficiently complete to permit the diagnosis of a new genus and species of this diverse clade. This discovery also clears up some of the confusion regarding the taxonomic composition of the pterosaur assemblage from the Kem Kem and helps demonstrate the presence of at least four distinct taxa. (1) An ornithocheirid (*Coloborhynchus moroccensis*) represented by a rostral fragment [16], a second as yet undescribed jaw fragment from Boumerade (collected by NI, SZ, LB) in the collections of the FSAC, and numerous isolated teeth [14], [15], [64]. (2) An azhdarchid (*Alanqa saharica*), detailed above. (3) A second edentulous taxon, represented by a single jaw fragment [15] that is likely tapejarid, or possibly thalassodromid. (4) A third edentulous taxon (represented by MN 7054-V and several additional undescribed specimens collected in Boumerade) with a relatively deep rostrum that has been identified as pteranodontid [22], but whose identity remains unclear. Further study, and ideally more material, is needed to fully resolve the identity of taxon three and four but, even without this resolution, it seems that at least three and possibly even four clades of pterosaurs are present in the Kem Kem beds (contra Averianov [21]). This pterosaur assemblage is thus the most diverse yet known from Africa; all others, with the exception of that from Tendaguru [2] and the Elrhaz Formation of Niger [12] are monospecific.

The extent to which some, or possibly all, of the Kem Kem pterosaur taxa were contemporaneous is not yet clear. However, the discovery of multiple specimens almost certainly representing multiple individuals (for all but the tapejarid/thalassodromid) of differing sizes, suggests that these pterosaurs were part of the Kem Kem terrestrial vertebrate communities, and not accidental allochthonous occurences. The fossils collected so far demonstrate considerable variation in rostral morphology. This hints at some diversity in feeding ecology, since rostral morphology seems to be closely related to feeding ecology in pterosaurs (e.g. [65]), and might indicate niche partitioning.

### Azhdarchid evolutionary history

The identification of *Alanqa* in the Kem Kem assemblage significantly increases our knowledge and understanding of azhdarchid evolutionary history, in particular, and of pterosaurs as a whole. The vast majority of azhdarchid fossils are of late Upper Cretaceous (Campanian-Maastrichtian) age and it seems that, in addition to being far more speciose than any other clade during this interval, azhdarchids were also morphologically and ecologically diverse during this time [65]. Azhdarchids have also been reported from the early Upper Cretaceous (Cenomanian-Santonian), but the record from this interval is much poorer and largely based on fragmentary finds from the Cenomanian and Turonian of Uzbekistan [44], [66], [67], [68]. *Alanqa*, and other finds from the Kem Kem that have been attributed to azhdarchids [25], [63], provide the best evidence to date for the presence of Azhdarchidae in the Cenomanian.

This conclusion is consistent with recent phylogenetic analyses [28], which predict that this clade first arose in the Early Cretaceous or possibly even earlier. Sayão and Kellner [69] identified an elongate cervical vertebra from the Upper Jurassic Tendaguru beds as azhdarchid, but this has been contradicted by Andres and Ji [70] who identified this find as ctenochasmatoid. This reassignment is supported by the close similarity of the Tendaguru vertebra to those of the gnathosaurine ctenochasmatoid ‘*Pterodactylus*’ *longicollum* [71]. Several Lower Cretaceous finds, including a partial skeleton from the Crato Formation [72], [73] and a humerus from the Glen Rose Formation [74], have also been assigned to the Azhdarchidae. However, in a recent review Unwin and Martill [75] identified the Crato specimen as probably tapejarid, and we note that the Glen Rose humerus, while possibly azhdarchoid, does not exhibit any unique features allying it with azhdarchids.

The temporal significance of *Alanqa* is emphasised by the geographic location of the finds. Amost all early Upper Cretaceous records of azhdarchids are from Laurasia (e.g. [68]). *Alanqa* demonstrates that azhdarchids had already spread to Gondwana by the beginning of the Late Cretaceous. This idea is supported by other younger records [8], [76] and suggests that azhdarchids had already achieved a wide distribution by the early Late Cretaceous.

The Kem Kem finds together with those from the early Upper Cretaceous of Middle Asia (e.g. [67]) also demonstrate that very large size (wingspans of six meters, or more) and unique features of the skull (long, straight rostra), cervical vertebrae (extreme elongation, complete confluency of the neural arch and centrum), and wing phalanges (ventral ridge on wing-phalanges two and three) were present at an early stage in azhdarchid evolutionary history and not restricted to late Upper Cretaceous taxa. This, in turn, suggests that the evolution of very large and giant size was not a simple linear progression in azhdarchids, but much more complex, with size reduction (e.g., *Azhdarcho* [44], *Montanazhdarcho* [77]) as well as size increase (*Quetzalcoatlus* [45], *Arambourgiania* [60]). In addition, it may also be that the appearance of ‘unusual’ morphologies such as that evident in the mandibular symphsis of *Bakonydraco* [48], which in some respects is more reminiscent of that of other azhdarchoids, rather than azhdarchids such as *Alanqa*, *Azhdarcho*, *Quetzalcoatlus* and *Zhejiangopterus*, may be secondarily derived within Azhdarchidae, rather than evidence of a late surviving relatively plesiomorphic lineage. Again this hints at some complexity in azhdarchid evolution.

### Nomenclatural Acts

The electronic version of this document does not represent a published work according to the International Code of Zoological Nomenclature (ICZN), and hence the nomenclatural acts contained in the electronic version are not available under that Code from the electronic edition. Therefore, a separate edition of this document was produced by a method that assures numerous identical and durable copies, and those copies were simultaneously obtainable (from the publication date noted on the first page of this article) for the purpose of providing a public and permanent scientific record, in accordance with Article 8.1 of the Code. The separate print-only edition is available on request from PLoS by sending a request to PLoS ONE, 185 Berry Street, Suite 3100, San Francisco, CA 94107, USA along with a check for $10 (to cover printing and postage) payable to “Public Library of Science”.

In addition, this published work and the nomenclatural acts it contains have been registered in ZooBank, the proposed online registration system for the ICZN. The ZooBank LSIDs (Life Science Identifiers) can be resolved and the associated information viewed through any standard web browser by appending the LSID to the prefix “http://zoobank.org/”. The LSID for this publication is: urn:lsid:zoobank.org:pub:3C8E93FF-E070-46BE-B123-4E64E86B520E

## References

[1] Reck H (1931). Die deutschostafrikanischen Flugsaurier.. Centralblatt f Min Geol u Pal.

[2] Unwin DM, Heinrich WD (1999). On a pterosaur jaw from the Upper Jurassic of Tendaguru (Tanzania).. Mitt Mus Nat.kd Berl, Geowiss Reihe.

[3] Aberhan M, Bussert R, Heinrich W-D, Schrank E, Schultka S (2002). Palaeoecology and depositional environments of the Tendaguru Beds (Late Jurassic to Early Cretaceous, Tanzania).. Mitt Mus Nat.kd Berl, Geowiss Reihe.

[4] Swinton WE (1984). A Cretaceous pterosaur from the Belgian Congo.. Bull Soc Belge Geol Paléont Hydr Liège.

[5] Dal Sasso C, Pasini G (2003). First record of pterosaurs (Pterosauria, Diapsida, Archosauromorpha) in the Middle Jurassic of Madagascar.. Atti Soc Ital Sci Nat Mus Civ Stor Nat Milano.

[6] Ntamak-Nida MJ, Ketchemen-Tandia B, Ewane RV, Lissock JP, Courville P (2006). Nouvelles données sur les Mollusques et autres macro-organismes campaniens de Sikoum (Centre-Est du sous basin de Douala-Cameroun): interérêts bio-chronologiques et paléo-écologiques.. Afr Geosc Rev.

[7] Blackbeard M, Yates AM (2007). The taphonomy of an Early Jurassic dinosaur bonebed in the northern Free State (South Africa).. J Vert Paleontol.

[8] Monteillet J, Lappartient JR, Taquet P (1982). Un Ptérosaurien géant dans le Crétacé supérieur de Paki (Sénégal).. C R Acad Sci II.

[9] Sereno PC, Beck AL, Dutheil DB, Gado B, Larsson HCE (1998). A long-snouted predatory dinosaur from Africa and the evolution of spinosaurids.. Science.

[10] Benton MJ, Bouaziz S, Buffetaut E, Martill DM, Ouaja M, Soussi M, Trueman C (2000). Dinosaurs and other fossil vertebrates from fluvial deposits in the Lower Cretaceous of southern Tunisia.. Palaeogeogr Palaeocl.

[11] Knoll F (2000). Pterosaurs from the Lower Cretaceous (?Berriasian) of Anoual, Morocco.. Ann Pal.

[12] Blackburn D (2002). Two Early Cretaceous pterosaurs from Africa.. J Vert Paleontol.

[13] Pereda-Suberbiola X, Bardet N, Jouve S, Iarochène M, Bouya B, Buffetaut E, Mazin JM (2003). A new azhdarchid pterosaur from the Late Cretaceous phosphates of Morocco.. Evolution and Palaeobiology of Pterosaurs.

[14] Mader BJ, Kellner AWA (1997). First occurrence of Anhangueridae (Pterosauria, Pterodactyloidea) in Africa.. J Vert Paleontol.

[15] Wellnhofer P, Buffetaut E (1999). Pterosaur remains from the Cretaceous of Morocco.. Palaeontol Z.

[16] Mader BJ, Kellner AWA (1999). A new anhanguerid pterosaur from the Cretaceous of Morocco. Bol. Museu Nacional ser.. Geologia.

[17] Owen R (1874). Monograph on the Fossil Reptilia of the Mesozoic Formations.. Monographs of the Palaeontographical Society.

[18] Kellner AWA, Tomida Y (2000). Description of a new species of Anhangueridae (Pterodactyloidea) with comments on the pterosaur fauna from the Santana Formation (Aptian-Albian), northeastern Brazil.. National Science Museum Monographs Tokyo.

[19] Unwin DM, Buffetaut E, Mazin JM (2003). On the phylogeny and evolutionary history of pterosaurs.. Evolution and Palaeobiology of Pterosaurs..

[20] Bennett SC (2001). The osteology and functional morphology of the Late Cretaceous pterosaur *Pteranodon*. Part I. General description of osteology.. Palaeontogr Abt A.

[21] Averianov AO, Arkhangelsky MS, Pervushov EM (2008). A new Late Cretaceous azhdarchid (Pterosauria, Azhdarchidae) from the Volga Region.. Paleontolog J.

[22] Kellner AWA, Mello AMS, Ford T (2007). A survey of pterosaurs from Africa with the description of a new specimen from Morocco..

[23] Lü JC, Jin X, Unwin DM, Zhao L, Azuma Y (2006). A new species of *Huaxiapterus* (Pterosauria: Pterodactyloidea) from the Lower Cretaceous of Western Liaoning, China with comments on the systematics of tapejarid pterosaurs.. Acta Geol Sin.

[24] Witton MP (2009). A new species of *Tupuxuara* (Thalassodromidae, Azhdarchoidea) from the Lower Cretaceous Santana Formation of Brazil, with a note on the nomenclature of Thalassodromidae.. Cretaceous Res.

[25] Kellner AWA, Mader BJ (1996). First report of Pterosauria (Pterodactyloidea, Azhdarchidae) from Cretaceous rocks of Morocco.. J Vert Paleontol.

[26] Vullo R, Neraudeau D (2009). Pterosaur remains from the Cenomanian (Late Cretaceous) Paralic Deposits of Charentes, Western France.. J Vert Paleontol.

[27] Lü JC, Unwin DM, Xu L, Zhang X (2008). A new azhdarchoid pterosaur from the Lower Cretaceous of China and its implications for pterosaur phylogeny and evolution.. Naturwissenschaften.

[28] Lü JC, Unwin DM, Jin X, Liu Y, Ji Q (2009). Modular evolution in a long-tailed pterosaur with a pterodactyloid skull.. Proc R Soc B: Biol Sci.

[29] Lavocat R (1949). Les gisements de vertébrés crétacés du sud Marocain.. C R Acad Sci.

[30] Lavocat R (1954). Reconnaissance géologique dans les Hammadas des confins algéro-marocains du sud. Notes Mem. Serv. geol.. Maroc.

[31] Lefranc JP, Guiraud R (1990). The Continental Intercalaire of northwestern Sahara and its equivalents in the neighbouring regions.. J Afr Earth Sci.

[32] Sereno PC, Dutheil DB, Iarochene M, Larsson HCE, Lyon GH (1996). Predatory dinosaurs from the Sahara and Late Cretaceous faunal differentiation.. Science 272:.

[33] Russell DA, Taquet P (1998). New data on spinosaurid dinosaurs from the Early Cretaceous of the Sahara.. C R Acad Sci Ser IIa: Sci Terre Planetes.

[34] Buffetaut E (1994). A new crocodilian from the Cretaceous of Southern Morocco.. C R Acad Sci 319.

[35] Russell DA (1996). Isolated dinosaur bones from the Middle Cretaceous of the Tafilalt, Morocco.. Bull Mus Natn Hist Nat Paris C.

[36] Dal Sasso C, Maganuco S, Buffetaut E, Mendez MA (2005). New information on the skull of the enigmatic theropod *Spinosaurus* with remarks on its size and affinities.. J Vert Paleontol.

[37] Larsson HCE, Sues HD (2007). Cranial osteology and phylogenetic relationships of *Hamadasuchus rebouli* (Crocodyliformes: Mesoeucrocodylia) from the Cretaceous of Morocco.. Zool J Linn Soc.

[38] Ferrandini M, Philip J, Babinot JF, Ferrandini J, Tronchetti G (1985). La plate-forme carbonatée du Cénomano-Turonien de la région d'Erfoud-Errachidia (Sud-Est marocain): stratigraphie et paléoenvironnements.. Bull Soc Geol Fr.

[39] Catuneanu O, Khalifa MA, Wanas HA (2006). Sequence stratigraphy and incised-valley systems of the Cenomanian Bahariya Formation, Western Desert, Egypt.. Sediment Geol.

[40] Cavin L, Dutheil DB (1999). A new Cenomanian ichthyofauna from southeastern Morocco and its relationships with other early Late Cretaceous Moroccan faunas.. Geol Mijnbouw.

[41] Unwin DM (2001). An overview of the pterosaur assemblage from the Cambridge Greensand (Cretaceous) of Eastern England. Mitt Mus Nat.kd.. Berl, Geowiss Reihe.

[42] Kaup JJ (1834). Versuch einer Eintheilung der Saugethiere in 6 Stämme und der Amphibien in 6 Ordnungen.. Isis.

[43] Plieninger F (1901). Beiträge zur Kenntnis der Flugsaurier.. Paläontogr.

[44] Nesov LA (1984). Upper Cretaceous pterosaurs and birds from Central Asia.. Paleontol Zh.

[45] Kellner AWA, Langston W (1996). Cranial remains of *Quetzalcoatlus* (Pterosauria, Azhdarchidae) from Late Cretaceous sediments of Big Bend National Park.. J Vert Paleontol.

[46] Cai Z, Wei F (1994). On a new pterosaur (*Zhejiangopterus linhaiensis* gen. et sp. nov.) from Upper Cretaceous in Linhai, Zhejiang, China.. Vertebrat Palasiatic.

[47] Owen R (1859). Monograph on the fossil Reptilia of the Cretaceous Formations.. Supplement No. I. Pterosauria (*Pterodactylus*).

[48] Ösi A, Weishampel DB, Jianu CM (2005). First evidence of azhdarchid pterosaurs from the Late Cretaceous of Hungary.. Acta Palaeontol Pol.

[49] Frey E, Tischlinger H, Buchy M-C, Martill DM, Buffetaut E, Mazin J.-M (2003). New specimens of Pterosauria (Reptilia) with soft parts with implications for pterosaurian anatomy and locomotion.. Evolution and Palaeobiology of Pterosaurs.

[50] Bakhurina NN, Unwin DM (1995). A survey of pterosaurs from the Jurassic and Cretaceous of the former Soviet Union and Mongolia.. Hist Biol.

[51] Bennett SC (2003). New crested specimens of the Late Cretaceous pterosaur *Nyctosaurus*.. Paläont Z.

[52] Frey E, Buchy MC, Stinnesbeck W, González AG, Stefano A (2006). *Muzquizopteryx coahuilensis* n.g., n. sp., a nyctosaurid pterosaur with soft tissue preservation from the Coniacian (Late Cretaceous) of northeast Mexico) (Coahuila).. Oryctos.

[53] Kellner, AWA (2004). New information on the Tapejaridae (Pterosauria, Pterodactyloidea) and discussion of the relationships of this clade.. Ameghiniana.

[54] Wellnhofer P, Kellner AWA (1991). The skull of *Tapejara wellnhoferi* Kellner (Reptilia: Pterosauria) from the Lower Cretaceous Santana Formation of the Araripe Basin, Northeastern Brazil.. Mitt Bayer Staat Paläontol histor Geol.

[55] Wang X, Zhou Z (2003). Two new pterodactyloid pterosaurs from the Early Cretaceous Jiufotang Formation of Western Liaoning, China.. Vertebrat Palasiatic.

[56] Witton MP (2008). A new azhdarchoid pterosaur from the Crato Formation (Lower Cretaceous, Aptian?) of Brazil.. Palaeontology.

[57] Kellner AWA, Campos D de A (2002). The function of the cranial crest and jaws of a unique pterosaur from the early Cretaceous of Brazil.. Science.

[58] Unwin DM, Lü JC (1997). On *Zhejiangopterus* and the relationships of pterodactyloid pterosaurs.. Hist Biol.

[59] Buffetaut E, Laurent Y, Le Loeuff J, Bilotte M (1997). A terminal Cretaceous giant pterosaur from the French Pyrenees.. Geol Mag.

[60] Frey E, Martill DM (1996). A reappraisal of *Arambourgiania* (Pterosauria, Pterodactyloidea): one of the world's largest flying animals.. Neues Jahrb Geol P-M.

[61] Bogolubov NN (1914). On the vertebra of a pterodactyl from the Upper Cretaceous beds of Saratoff Province (in Russian).. Ann Geol Min Russia.

[62] Martill DM, Frey E, Sadaqah RM, Khoury HN (1998). Discovery of the holotype of the giant pterosaur *Titanopteryx philadelphiae* Arambourg 1959, and the status of *Arambourgiania* and *Quetzalcoatlus*.. N Jb Geol Paläont Abh.

[63] Rodrigues T, Kellner AWA, Mader B, Russell D (2006). Brief report on new pterosaur (Pterosauria, Pterodactyloidea) specimens from the Cretaceous of Morocco.. J Vert Paleontol.

[64] Kellner AWA, Mader BJ (1997). Archosaur teeth from the Cretaceous of Morocco.. J Paleontol.

[65] Witton MP, Naish D (2008). A reappraisal of azhdarchid pterosaur functional morphology and paleoecology.. PLoS ONE.

[66] Averianov, AO (2004). New data on Cretaceous flying reptiles (Pterosauria) from Russia, Kazakhstan, and Kyrgystan.. Paleontolog J.

[67] Averianov AO (2007). New records of azhdarchids (Pterosauria, Azhdarchidae) from the Late Cretaceous of Russia, Kazakhstan and Central Asia.. Paleontolog J.

[68] Barrett PM, Butler RJ, Edwards NP, Milner AR (2008). Pterosaur distribution in time and space: an atlas.. Zitteliana B.

[69] Sayão JM, Kellner AWA (2001). Comments on pterosaur fauna from Tendaguru, Upper Jurassic of Africa, with the identification of a possible Azhdarchid..

[70] Andres B, Ji Q (2008). A new pterosaur from the Liaoning Province of China, the phylogeny of the Pterodactyloidea, and convergence in their cervical vertebrae.. Palaeontology.

[71] Plieninger, F (1907). Die Pterosaurier der Juraformation Schwabens.. Paläontogr.

[72] Martill DM, Frey E (1998). A possible azhdarchid pterosaur from the Crato Formation (Early Cretaceous, Aptian) of Brazil.. Abstract Third European Workshop on Vertebrate Palaeontology, Maastricht.

[73] Martill DM, Frey E (1999). A possible azhdarchid pterosaur from the Crato Formation (Early Cretaceous, Aptian) of northeast Brazil.. Geol Mijnb.

[74] Murray PA, Winkler DA, Jacobs LL (1991). An azhdarchid pterosaur humerus from the Lower Cretaceous Glen Rose Formation of Texas.. J Paleontol.

[75] Unwin DM, Martill DM, Martill DM, Bechly G, Loveridge RF (2007). Pterosaurs from the Lower Cretaceous Crato Formation of the Chapada do Araripe, north east Brazil.. The Fossils of the Crato Formation: Window on an Ancient World.

[76] Codorniú L, Gasparini Z, Gasparini Z, Salgado L, Coria RA (2007). Pterosauria.. Patagonian Mesozoic Reptiles.

[77] Padian K, Horner JR, de Ricqlès AJ (1993). A new azhdarchid pterosaur from the Two Medicine Formation (Late Cretaceous, Campanian) of Montana, identified on the basis of bone histology.. J Vert Paleontol.

